# Comparative Analysis of Early-Stage Clinical Features Between COVID-19 and Influenza A H1N1 Virus Pneumonia

**DOI:** 10.3389/fpubh.2020.00206

**Published:** 2020-05-15

**Authors:** Changxing Shen, Min Tan, Xiaolian Song, Guoliang Zhang, Jiren Liang, Hong Yu, Changhui Wang

**Affiliations:** ^1^Department of Pulmonary and Critical Care Medicine, Shanghai Tenth People's Hospital, School of Medicine, Tongji University, Shanghai, China; ^2^Disease and Infection Control Office, Shanghai Tenth People's Hospital, School of Medicine, Tongji University, Shanghai, China

**Keywords:** coronavirus disease 2019, Influenza A, Pneumonia, early stage, Clinical features, grid-form shadow

## Abstract

**Introduction:** Influenza virus pneumonia and COVID-19 are two different types of respiratory viral pneumonia but with very similar clinical manifestations. The aim of the present study was to help clinicians gain a better understanding about differences between Influenza virus pneumonia and COVID-19 by comparative analysis of the early-stage clinical features.

**Methods:** Clinical data of patients with confirmed diagnosis of COVID-19 and influenza A pneumonia identified in our hospital were collected and analyzed retrospectively to identify the clinical features that could differentiate between the two types of viral pneumonia.

**Results:** The two types of viral pneumonia mainly affected adults, especially people over 50 years, with no gender difference between them. Fever, cough, sputum and muscle soreness were the most common symptoms of COVID-19. Some patients with COVID-19 may also exhibit digestive tract symptoms. Elevation of C-reactive protein (CRP) was a more common phenomenon in patients with COVID-19 than that in patients with influenza A H1N1 virus pneumonia. In addition, eosinophil count was decreased and the monocyte percentage was increased in COVID-19 patients. The grid-form shadow was a typical presentation of COVID-19 on the lung CT image, and the disease usually progressed quickly within a week.

**Conclusion:** Influenza pneumonia and COVID-19 are two different types of respiratory viral pneumonia with very similar clinical manifestations. The percentage of monocytes is increased and the eosinophil count is decreased in COVID-19. Glass-ground density exudation shadow located peripherally is the typical sign of COVID-19 on the lung CT image, and the shadow often with grid-form sign. These features may not be typically observed in patients with influenza pneumonia. Chest CT scan combined with nucleic acid detection is an effective and accurate method for diagnosing COVID-19. Blood routine test has a limited diagnostic value in differentiating the two forms of pneumonia.

## Introduction

2019 novel coronavirus (2019-ncov) bring about a great threat to human health. Clinicians in China have successfully dealt with the epidemic and accumulated rich clinical experience in the diagnosis and treatment of COVID-19. During the winter and spring seasons, influenza virus pneumonia is a common disease. A considerable number of influenza virus infections have occurred in the United States. Influenza pneumonia and COVID-19 are two different forms of respiratory viral pneumonia but share similar clinical manifestations. In addition, there is even the chance of contracting a mixed infection of both influenza virus and COVID-19 simultaneously, and therefore it is very important to make a differential diagnosis between them. In this article, we tried to summarize the typical clinical features of COVID-19 and influenza A virus H1N1 pneumonia and present a comparative analysis to enable clinicians to gain a better understanding about the differences between the two different forms of respiratory virus-induced pneumonia.

## Materials and Methods

The clinical data of 15 patients with COVID-19 etiologically confirmed in our hospital between January 22 and February 20, 2020 were analyzed retrospectively. The diagnostic criteria were implemented by referring to the Chinese New Coronary Virus Pneumonia Diagnosis and Treatment Program (Trial Version 4), and all patients with COVID-19 were excluded from influenza A/B pneumonia by influenza virus antigen test. Additionally, the clinical data of 18 patients with influenza A H1N1 virus pneumonia who received treatment in the respiratory department of the same hospital between the winter of 2018 and the spring of 2019 were collected for comparative study. The diagnosis was confirmed by a combination of nasopharyngeal secretion influenza virus antigen detection and the clinical manifestations. The criteria for determining the epidemiological history of influenza virus (China's influenza diagnosis and treatment program - 2018 version) are as follows: Influenza patients and recessive infected persons are the main sources of influenza infection, and they are infectious from the end of the latent period throughout the acute period. Infected animals may also become the source of infection. Doctors need to ask if they have close contacts with infected persons. Generally, the time for the infected person to release the virus is 3–6 days, and the time for some people with relatively low immune function to release the virus can be as long as 1–3 weeks. The symptoms, medical history and initial laboratory test findings including routine blood tests, C-reactive protein (CRP) level, influenza A/B antigen results, chest CT imaging findings and 2019 novel coronavirus nucleic acid test of these patients were collected after disease onset. Nasopharyngeal secretion specimens were gathered directly by the attending physicians. The routine blood test, CRP test and influenza antigen test were all performed by the Department of Laboratory Medicine of Shanghai Tenth People's Hospital, using the normal reference values as follows: white blood cell (WBC) count: 3.5–9.5 × 10^9^/L; neutrophil count: 1.8–6.3 × 10^9^/L; neutrophil percentage: 40–75%; monocyte count: 0.1–0.6 × 10^9^/L; monocyte percentage: 3%-10%; lymphocyte count: 1.1–3.2 × 10^9^/L; lymphocyte percentage: 20–50%; eosinophil count: 0.02–0.52 × 10^9^/L; platelet count: 125–350 × 10^9^/L; and CRP: < 8.2 mg/L. Influenza antigen detection using influenza A/B virus antigen detection reagents (i.e., Colloidal gold method) was conducted by Guangzhou Wondfo Biotech Co., Ltd (Guangzhou, China). A chest CT plain scan was completed in a specialized CT room by the Department of Radiology of the said hospital. The examination reports were written and reviewed by a senior radiologist. The 2019-nCoV nucleic acid detection process adopted a quantitative real-time polymerase chain reaction (qPCR) method. All the pathogenic examination methods were according to the Chinese New Coronary Virus Pneumonia Diagnosis and Treatment Program (Trial Version 4), including collecting secretion samples from the nasopharynx, using blood samples from patients with fever or sputum samples from patients with pneumonia, or stool samples from patients with digestive tract symptoms, and using the kit to test the nucleic acid of the samples. Specimen collection and the report release were completed by a representative from Shanghai Municipal Center for Disease Control & Prevention. The 15 confirmed cases of COVID-19 were defined as COVID-19 group, and the 18 cases of influenza A virus pneumonia were defined as influenza A group. The clinical characteristics of the two pneumonia groups were analyzed and compared to establish differences. The study was reviewed and approved by the Ethics Committee of Tongji University Tenth People's Hospital (Approval No: SHSY - IEC - 4.1/20 - 23/01).

### Statistics

Using the SPSS version 23.0 software program (IBM Corp., Armonk, NY, USA), normal distribution measurement data are expressed as x ± s. Comparison of the mean values between the two groups was performed by a *t*-test, and comparison of more than three mean values was performed by a variance test. Non-normal distribution measurement data were described by median using the rank-sum test. Count data were analyzed using the chi-square test. A *p* < 0.05 was considered to be statistically significant. Relevant diagnostic variables were firstly subjected to single-factor logistic regression analysis, and the statistically significant factors of single-factor regression were adopted subsequently as independent variables for logistic regression analysis.

## Results

**Figure 1:**
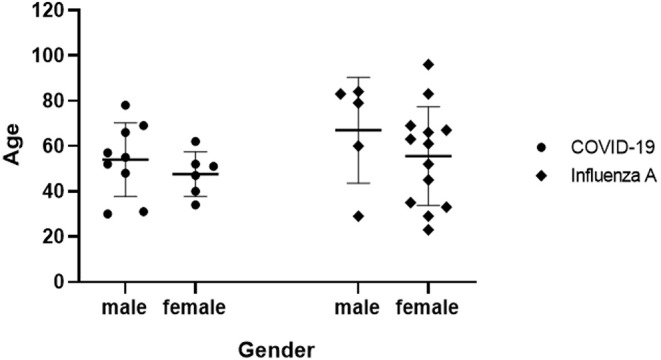
A nested graph: gender and age range of patients in COVID-19 group and Influenza A group.

**Figure 2:**
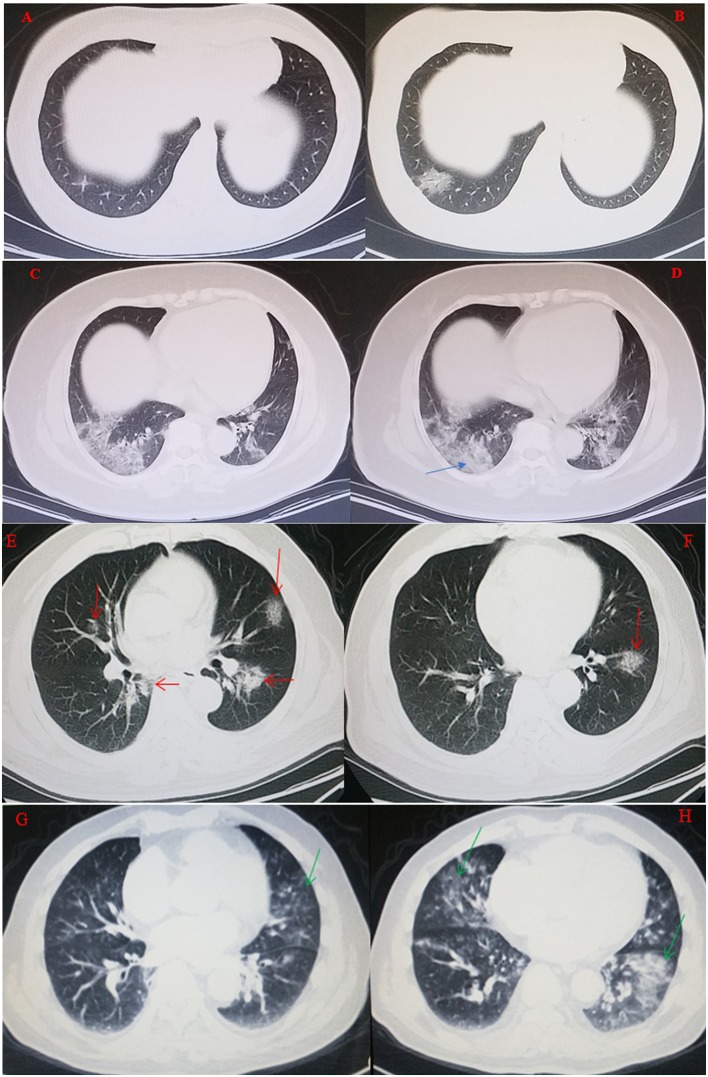
A 48-year-old woman visited the hospital due to fever, cough, and a history of traveling to Wuhan, China for 3 days. She underwent a chest CT scan on January 22, 2020, which showed a lesion pattern with the density of ground glass in the outside lung field of the right lower lung, **(A)**. She was diagnosed with COVID-19 on January 23, 2020 and with a chest CT review performed on January 24, 2020, it can be seen that the original lesion pattern had progressed to a round state, **(B)**. A 62-year-old woman visited the hospital due to cough, dyspnea, and fever for 4 days. There was no clear history of contact with an infected person. On January 28, 2019, a chest CT scan suggested a lesion pattern with the density of ground glass in the peripheral lungs, **(C)**. She was diagnosed with influenza A pneumonia on January 30, 2019. Chest CT findings were reviewed again on January 31, at which point, the lesions had progressed with consolidations, as indicated by the arrows, and appeared patchy, **(D)**. A 67 year old male patient with chest tightness and cough for 5 days, underwent lung plain CT scan on January 31, 2020. CT image: multiple ground glass density exudation shadow with grid shadow in both lungs, as the arrow points out **(E,F)** are CT images at the same day, with different scan levels. Nasopharyngeal swab for 2019-nCoV nucleic acid test positive. A 61-year-old male patient with a cough and fever for 3 days, on Febuary 1, 2019, lung pain scan CT was examined, ground glass density exudation shadow distributed in multiple parts of two lungs, as the arrow points out, **(G,H)** ae CT images at the same day, with different scan levels. Nasopharyngeal swab tested positive for influenza A antigen.

The 15 patients in COVID-19 group ranged in age from 30 to 78 years with a median of 52 years, and the 18 patients in influenza A group ranged in age from 23 to 96 years with a median of 62 years, Figure 1 provides the details of male and female age range in COVID-19 and influenza virus infected persons. Table 1 presents the data from the two study groups, there was no significant difference between the two groups in mean age, sex ratio, main symptoms, WBC count, lymphocyte count, monocyte count, latelet count, CRP level, lesion distribution on chest CT scans, onset time. However, the history of epidemiological exposure, neutrophil count, neutrophil percentage, lymphayte percentage, monocyte percentage, eosinophil count, comorbidities (except for chronic kidney disease, chronic digestive disease, autoimmune disease, tumor) and disease evolution findings of the two groups were significant. In univariate logistic regression, the diagnosis of COVID-19 or influenza A pneumonia was adopted as the dependent variable, while related symptoms, the significant factors, such as, epidemiological history and some laboratory results were chosen as independent variables. The univariate logistic regression was performed to clearly distinguish the main disease factors of COVID-19 and influenza A pneumonia. Ultimately, an epidemiological exposure history, neutrophil percentage < 60%, lymphocyte percentage < 20% and eosinophil count < 0.01 × 10^9^/L were identified as the four main significant factors to distinguish the diagnosis between COVID-19 and influenza A pneumonia, the specific data is shown in Table 2. The results of multivariate logistic regression are shown in Table 3. Eosinophil count < 0.01 × 10^9^/L and a clear history of epidemiological exposure were used as two statistically significant factors to distinguish COVID-19 from influenza A pneumonia. CT imaging characteristics of the two forms of viral pneumonia are presented in Table 4. It was found that early ground-glass density exudation in the lung, a consolidation of lesions occurring during disease progression, distribution of the lesions mainly outside the lung, and less frequent appearance of pleural effusion were common signs for both COVID-19 and influenza A pneumonia. The grid-form shadow was a typical significant sign for the diagnosis of COVID-19 in the early stage. Figure 2 through 5 are typical CT images of two COVID-19 cases and two influenza A pneumonia cases.

**Table 1:** Characteristics of the two respiratory viral pneumoniae groups.

**Characteristics**	**COVID-19 group(*n* = 15)**	**Influenza A group (*n* = 18)**	***p*-value**
Number	15	18	
Age (years)	51.07 ± 13.20	58.72 ± 22.19	0.23
Gender (male/female)	9/6	5/13	0.13
Epidemiological exposure history (yes/no)	12/3	4/14	0.00^*^
Onset time	3.63 ± 1.01	3.90 ± 1.22	0.69
Temperature (°C)	38.1 ± 0.65	38.1 ± 1.03	0.98
Headache (yes/no)	3/12	2/16	0.8
Diarrhea (yes/no)	1/14	0/18	0.9
Vomit (yes/no)	0/15	1/17	1
Dyspnea (yes/no)	1/14	4/14	0.3
Muscular soreness (yes/no)	11/4	10/8	0.46
Chest pain (yes/no)	1/14	0/18	0.45
Pharyngalgia (yes/no)	6/9	5/13	0.48
Rhinorrhea (yes/no)	6/9	4/14	0.45
Cough and expectoration (yes/no)	13/2	16/2	1
CRP level (mg/L)	19.55 ± 2.39	17.26 ± 4.52	0.08
WBC count (×10^9^/L)	6.04 ± 2.44	7.94 ± 3.89	0.10
Neutrophil count	3.44 ± 0.26	5.67 ± 0.49	0.01^*^
Neutrophil percentage (%)	57.05 ± 10.97	71.46 ± 12.74	0.00^*^
Lymphocyte count (×10^9^/L)	2.03 ± 1.72	1.24 ± 0.54	0.1
Lymphocyte percentage (%)	32.13 ± 12.19	19.07 ± 11.47	0.00^*^
Monocyte count (×10^9^/L)	0.58 ± 0.23	0.58 ± 0.34	0.98
Monocyte percentage (%)	10.2 ± 3.26	7.85 ± 2.94	0.04^*^
Eosinophil count (×10^9^/L)	0.01 (0–0.13)	0.08 (0–0.28)	0.02^*^
Platelet count (×10^9^/L)	172.2 ± 47.58	227.61 ± 91.14	0.07
Hypertension (yes/no)	0/15	8/10	0.00^*^
Diabetes (yes/no)	0/15	5/13	0.05^*^
Cardio-cerebrovascular disease (yes/no)	0/15	5/13	0.05^*^
Chronic lung disease (yes/no)	0/15	6/12	0.02^*^
Chronic kidney disease (yes/no)	0/15	0/18	0.6
Chronic digestive disease (yes/no)	0/15	2/16	0.49
AID (yes/no)	1/14	5/13	0.19
Tumor (yes/no)	0/15	4/14	0.1
Involvement of lungs on chest CT (unilateral/bilateral)	8/7	9/9	1
Disease aggravation within 7 days (yes/no)	13/2	9/9	0.03^*^

n, number; COVID-19, Coronavirus disease 2019; AID, autoimmune disease; CRP, C-reactive protein; CT, computed tomography; WBC, white blood cell;

* *, Statistically significant*.

**Table 2:** Univariate regression analysis.

**Variable**	**Coefficient**	**95% CI**	***p*-value**
Epidemiological history	2.64	2.91–89.72	0.00^*^
Neutrophil percentage <60%	−2.30	0.02–0.47	0.00^*^
Lymphocyte percentage <20%	2.86	2.64–352.8	0.01^*^
Eosinophil count <0.01 × 10^9^/L	0.02	0.02–0.44	0.00^*^
CRP level >8 mg/L	−1.09	0.07–1.39	0.14

CRP, C-reactive protein; CI, confidence interval;

* *, Statistically significant*.

**Table 3:** Multivariate regression analysis.

**Variable**	**Coefficient**	**95% CI**	***p*-value**
Epidemiological history	3.13	3.27–440.5	0.01^*^
Neutrophil percentage <60%	−1.20	0.04–2.16	0.25
Lymphocyte percentage <20%	2.86	1.11–230.9	0.07
Eosinophil count <0.01 × 10^9^/L	−2.45	0.01–0.48	0.01^*^

CI, confidence interval;

* *, Statistically significant*.

**Table 4:** CT image characteristics of the two respiratory viral pneumoniae groups.

**CT image characteristics**	**COVID-19 group (*n* = 15)**	**Influenza A group (*n* = 18)**	**chi-square-value**	***p*-value**
**Lung lobe**				
Left upper lobe (yes/no)	10/5	3/15	8.58	0.03^*^
Left lower lobe (yes/no)	9/6	13/5	0.55	0.46
Right upper lobe (yes/no)	9/6	2/16	8.8	0.03^*^
Right middle lobe (yes/no)	10/5	3/15	8.57	0.03^*^
Right lower lobe (yes/no)	10/5	12/6	0	1
Lung periphery (yes/no)	13/2	16/2	0.38	0.85
**Number of lesions**				
Single lesion (yes/no)	2/13	3/15	0.71	0.79
Two lesions (yes/no)	2/13	2/16	0.38	0.85
Above two lesions (yes/no)	11/4	12/6	0.17	0.68
**CT signs**				
Nodular (yes/no)	5/10	3/15	1.24	0.27
Ground-glass opacity (yes/no)	14/1	18/0	–	0.46
Grid-form shadow (yes/no)	10/5	5/13	4.99	0.025^*^
Fibrotic streaks (yes/no)	12/3	10/8	2.2	0.14
Tree-in-bud (yes/no)	6/9	4/14	1.22	0.27
Air-bronchogram (yes/no)	7/8	3/15	3.48	0.06
Mediastinal lymphadenectasis (yes/no)	1/14	1/17	0.18	0.89
Pleurorrhea (yes/no)	1/14	1/17	0.18	0.89
Pleural thickening (yes/no)	7/8	6/19	0.61	0.43

n, number; CT, computed tomography; COVID-19, Coronavirus disease 2019;

* *, Statistically significant*.

## Discussion

An overview of the clinical data of the two forms of viral pneumonia reveals that they mainly occur in middle-aged to elderly adults, although both may also readily infect infants and young children, knowing that the immunity of these people is relatively lower than that of young adults. There is no significant difference in gender factors between the two forms of viral pneumonia (1, 2), although other studies reported that the male sex was more susceptible to COVID-19, and chronic comorbidities (which may increase all-cause mortality) were more frequent in male patients (3–5). Both diseases show person-to-person transmission characteristics. Per the data in this study, patients with influenza A pneumonia often had coexisting underlying diseases such as hypertension, diabetes, cardiocerebrovascular disease, and chronic lung disease, although there are also studies reporting coexisting diseases in COVID-19 patients (6). In general, patients with underlying diseases tend to be relatively immunocompromised and more susceptible to viral infection. From an epidemiological point of view, most patients with COVID-19 had a clear history of close contacts with infected persons or a history of staying in the affected area or had been in and out crowded areas in the COVID-19 epidemic area, such as shopping malls, airports, and railway stations (7, 8), COVID-19 is an emergency disease, showing a highly infectious and pathogenic nature. In investigating the history of epidemiology, we usually asked the patients very carefully, and even reviewed the videos of public places visited by infected people. But as we are not more familiar with influenza A pneumonia, we rarely tracked down the epidemiological exposure history, but this does not mean that people with influenza do not really have an epidemiological exposure history. Of the main symptoms of viral pneumonia reported in the early stage, fever, muscular soreness, cough, expectoration, pharyngalgia, and rhinorrhea are the most common, followed by gastrointestinal symptoms, such as dizziness, headache, diarrhea, nausea and vomiting (9). With progression of the disease, dyspnea and chest pain may appear. Some COVID-19 patients may not develop fever even though they may have progressed to severe pneumonia (10, 11). while patients with influenza A pneumonia are more prone to experiencing high fever. However, the incidence of gastrointestinal symptoms is higher in COVID-19 than that in influenza A virus (12), mainly because the digestive system is also the target organ of COVID-19. Analysis of the early laboratory examination findings showed that elevation of the CRP and the percentage of monocytes, and decrease of the eosinophil count are common characteristics of the two forms of viral pneumonia (9, 13, 14). But the mean value of neutrophil percentage and eosinophil count were significantly lower and the mean value of monocyte percentage was significantly higher in COVID-19 patients as compared with patients with influenza A virus (15). In the subsequent stage of viral infection, the leukocyte or neutrophil count may increase after combined bacterial infection, and the CRP level and procalcitonin content can similarly further rise. Notably, a simultaneous increase in neutrophil count and interleukin often indicates deterioration of the COVID-19 condition. The novel coronavirus is a kind of unprecedented infection, which can lead to excessive activation of the immune inflammatory response after infection (16–18), while the immune-induced inflammatory response will be further activated after bacterial infection, leading to an earlier inflammatory storm and resulting in acute respiratory failure and even multiple organ dysfunction. Thus, it is of great significance to monitor the routine blood test results of COVID-19 patients. On the CT images of both groups, the early lesions appeared with a density like that of ground glass in the lungs. With disease progression, the lesions appeared to consolidate or fuse. In the early stages of the disease (9, 14, 19, 20), the lesions were mainly distributed outside the lung, and pleural effusion was rarely seen. These four points are the common imaging manifestations of the two different forms viral pneumonia.

Lesions in COVID-19 pneumonia are more likely to appear in the upper lobe and right middle lobe, mostly in the form of a grid-form shadow. These features in COVID-19 may be the significant differences compared with influenza pneumonia. The specific mechanism driving this finding is not clear, and larger sample clinical studies are needed to confirm its validity. Data obtained from this study suggest that patients with COVID-19 are more likely to progress within 1 week (21, 22), and plain chest CT imaging is a very convenient means of monitoring the condition of the lung. We used disease diagnosis as the dependent variable, performed a univariate logistic regression, and then adopted univariate statistically significant variables for multivariate regression analysis. The results suggest that an eosinophil count < 0.01 × 10^9^/L and a clear epidemiological exposure history are primary significant factors that distinguish COVID-19 from influenza A pneumonia.

## Conclusion

Influenza pneumonia and COVID-19 are two different forms of respiratory viral pneumonia with very similar clinical manifestations. The monocyte percentage is increased and the eosinophil count is decreased in COVID-19. This change in WBC classification supports the diagnosis of early COVID-19. On CT images, COVID-19 pneumonia lesions mainly distribute outside the lung with ground-glass density exudation the form of a grid-form shadow. These features may be the significant differences that differentiate COVID-19 from influenza pneumonia. Chest CT examination combined with nucleic acid detection is an efficient and accurate method for the diagnosis of COVID-19.

## Data Availability Statement

All datasets generated for this study are included in the article/supplementary material.

## Ethics Statement

The studies involving human participants were reviewed and approved by Ethics Committee of Tongji University Tenth People's Hospital. The patients/participants provided their written informed consent to participate in this study.

## Author Contributions

CW contributed substantially to the study design. GZ, XS, JL, and HY contributed substantially to the data providing. CS had full access to all of the data in the study, took responsibility for the integrity of the data, and the accuracy of the data analysis including and especially any adverse effects, and contributed substantially to the writing of the manuscript. MT contributed substantially to the data analysis and interpretation.

## Conflict of Interest

The authors declare that the research was conducted in the absence of any commercial or financial relationships that could be construed as a potential conflict of interest.

## Abbreviations

COVID-19: coronavirus disease 2019
CT: computed tomography
nCoV: 2019 novel coronavirus
CRP: C-reactive protein
qPCR: quantitative real-time polymerase chain reaction
AID: autoimmune disease
WBC: white blood cell
CI: confidence interval.
